# Supplementary material for: MetaRibo-Seq measures translation in microbiomes
Source: Nat Commun. 2020 Jun 29;11:3268. doi: 10.1038/s41467-020-17081-z (PMC7324362; doi:10.1038/s41467-020-17081-z)
Supplement: Supplementary file 10 — Supplementary Data 7 [file 41467_2020_17081_MOESM10_ESM.zip › File2/Confidence_High_Taxonomy/345500_out.krona.html]

Javascript must be enabled to view this page.

members
magnitude
magnitudeUnassigned
count
unassigned
taxon
rank

345500\_out

40

40
2
superkingdom

40
976
phylum

40
200643
class

40
171549
order

815
family
1

816
genus
1


SRS023526\_contig\_number\_678
1
species
28116

39
family
171552

39
genus
838

species
59823
1

SRS013098\_contig\_number\_32915

species
165179
38

SRS011134\_contig\_number\_39620SRS012989\_contig\_number\_6545SRS013638\_contig\_number\_2078SRS015782\_contig\_number\_6071SRS015794\_contig\_number\_7792SRS017375\_contig\_number\_55SRS018888\_contig\_number\_2565SRS019808\_contig\_number\_14789SRS022609\_contig\_number\_2634SRS023526\_contig\_number\_17043SRS024132\_contig\_number\_23623SRS044535\_contig\_number\_2333SRS046502\_contig\_number\_13647SRS047044\_contig\_number\_13863SRS047044\_contig\_number\_17278SRS049712\_contig\_number\_21904SRS050752\_contig\_number\_20745SRS050752\_contig\_number\_21008SRS053398\_contig\_number\_1025SRS077194\_contig\_number\_11016SRS077641\_contig\_number\_2074SRS077991\_contig\_number\_55SRS078419\_contig\_number\_11677SRS097958\_contig\_number\_7774SRS104036\_contig\_number\_2344SRS1041134\_contig\_number\_9829SRS1041137\_contig\_number\_28152SRS104327\_contig\_number\_5967SRS104912\_contig\_number\_7073SRS105082\_contig\_number\_568SRS143876\_contig\_number\_23544SRS144297\_contig\_number\_10189SRS144506\_contig\_number\_44517SRS144603\_contig\_number\_5672SRS144714\_contig\_number\_9134SRS147614\_contig\_number\_5864SRS148253\_contig\_number\_4201SRS150029\_contig\_number\_14646
